# Specific Binding of Anionic Porphyrin and Phthalocyanine to the G-Quadruplex with a Variety of *in Vitro* and *in Vivo* Applications

**DOI:** 10.3390/molecules170910586

**Published:** 2012-09-05

**Authors:** Hidenobu Yaku, Takashi Murashima, Daisuke Miyoshi, Naoki Sugimoto

**Affiliations:** 1 Advanced Technology Research Laboratories, Panasonic Corporation, 3-4 Hikaridai, Seika-cho, Soraku-gun, Kyoto 619-0237, Japan; 2 Frontier Institute for Biomolecular Engineering Research (FIBER), Konan University, 7-1-20 Minatojima-minamimachi, Chuo-ku, Kobe 650-0047, Japan; Email: murasima@konan-u.ac.jp (T.M.); miyoshi@center.konan-u.ac.jp (D.M.); sugimoto@konan-u.ac.jp (N.S.); 3 Faculty of Frontiers of Innovative Research in Science and Technology (FIRST), Konan University, 7-1-20 Minatojima-minamimachi, Chuo-ku, Kobe 650-0047, Japan

**Keywords:** anionic phthalocyanine, DNAzyme, G-quadruplex, hemin, peroxidase, telomerase

## Abstract

The G-quadruplex, a four-stranded DNA structure with stacked guanine tetrads (G-quartets), has recently been attracting attention because of its critical roles *in vitro* and *in vivo*. In particular, the G-quadruplex functions as ligands for metal ions and aptamers for various molecules. Interestingly, the G-quadruplex can show peroxidase-like activity with an anionic porphyrin, iron (III) protoporphyrin IX (hemin). Importantly, hemin binds to G-quadruplexes with high selectivity over single-stranded DNA (ssDNA) and double-stranded DNA (dsDNA), which is attributable to an electrostatic repulsion of phosphate groups in ssDNA and dsDNA. The G-quadruplex and hemin-G-quadruplex complex allow development of sensing techniques to detect DNA, metal ions and proteins. In addition to hemin, anionic phthalocyanines also bind to the G-quadruplex formed by human telomere DNA, specifically over ssDNA and dsDNA. Since the binding of anionic phthalocyanines to the G-quadruplex causes an inhibition of telomerase activity, which plays a role in the immortal growth of cancer cells, anionic phthalocyanines are promising as novel anticancer drug candidates. This review focuses on the specific binding of hemin and anionic phthalocyanines to G-quadruplexes and the applications *in vitro* and *in vivo* of this binding property.

## 1. Introduction

**Figure 1:**
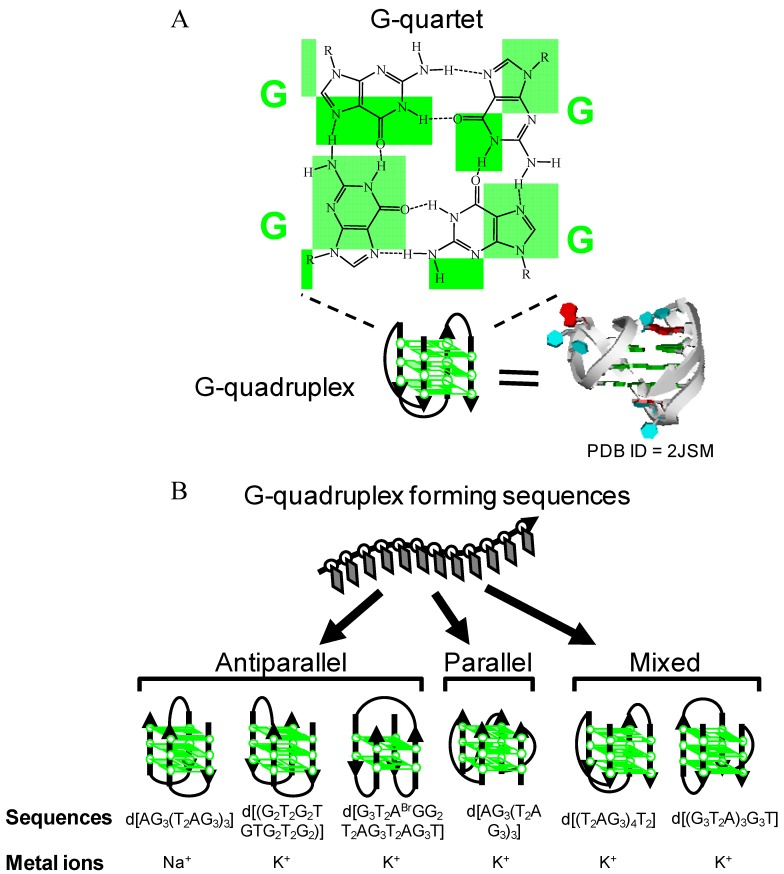
(**A**) Typical G-quadruplex and G-quartet structures. In the crystal structure of the G-quadruplex (right), the backbone is represented as a ribbon model. Thymine, adenine, and guanine bases are drawn in blue, red, and green, respectively; (**B**) Schematic diagram of relationship among sequences, metal ions and unimolecular G-quadruplex polymorphism.

**Figure 2:**
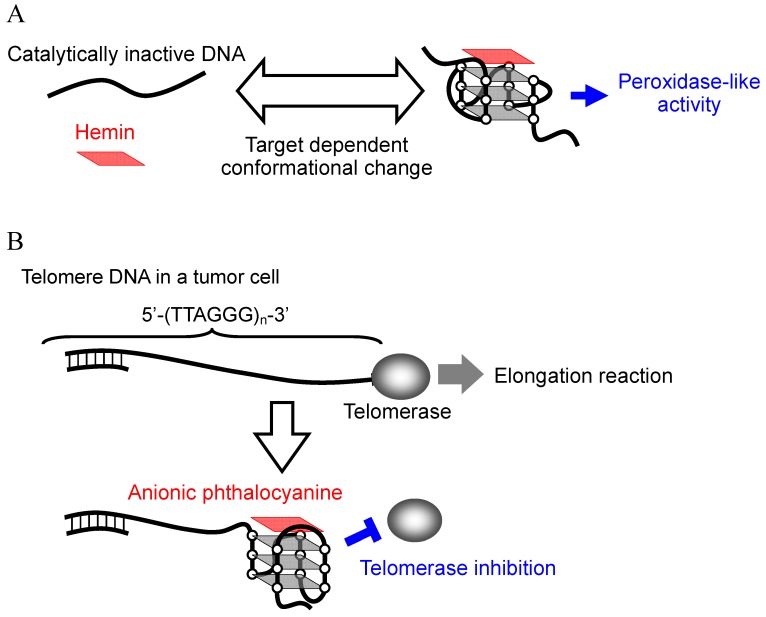
Schematic diagram illustrating the principle of the biosensing application utilizing hemin-G-quadruplex with peroxidase-like activity (**A**), and telomerase inhibition by anionic phthalocyanines (**B**).

The fabrication of functional nanomaterials, which includes functional dyes (porphyrins and phthalocyanines) [1,2,3], carbon nanomaterials (fullerenes, carbon nanotubes, or graphenes) [4,5,6] and nanoparticles (gold nanoparticles, magnetic nanoparticles, or quantum dots) [6,7,8,9], by biomolecules such as nucleic acids, proteins and lipids has recently been opening up an entire new and exciting research area. Among the biomolecules used, G-quadruplex DNA has been attracting attention because of its property of exhibiting peroxidase-like activity with iron (III) protoporphyrin IX, also known as hemin. The G-quadruplex is a four-stranded DNA structure with stacked guanine tetrads, G-quartets, which are held together via eight Hoogsteen hydrogen bonds (Figure 1A). Since the prediction of the existence of the G-quadruplex [10], G-quadruplexes formed by different guanine-rich (G-rich) sequences are found not only in genomic DNA but are also generated during *in vitro* screening for aptamers targeting molecules such as proteins [11,12,13,14]. Structural studies have demonstrated that the G-rich sequences can fold to form various types of G-quadruplex conformations depending on the sequences and the experimental conditions (e.g., coexisting metal ion, metal ion concentration and degree of molecular crowding) (Figure 1B) [11,12,13,14]. These findings combined with the peroxidase-like activity of the hemin-G-quadruplex complex have stimulated development of various applications, mainly detection techniques targeting DNA, proteins and metal ions [15,16,17,18]. The detection is composed of the following two steps. The first involves DNA conformational change to a catalytically active form of G-quadruplex from inactive DNA such as single-stranded DNA (ssDNA) and double-stranded DNA (dsDNA), depending on the target molecule. The second step is analysis of the peroxidase-like activity of the G-quadruplex with hemin (Figure 2A). The key point is the specific binding of hemin to the G-quadruplex because the system can not work if hemin binds to ssDNA and dsDNA nonspecifically and the complexes then exert peroxidase-like activity. Cationic p-conjugated molecules such as cationic porphyrins usually bind to G-quadruplexes via π-π interaction with G-quartets and via electrostatic interaction with anionic phosphate groups on G-quadruplexes [19,20,21,22,23,24,25,26]. However, the specificity of G-quadruplexes is usually low because the electrostatic interaction also causes non-specific binding to dsDNA and ssDNA [20,22,23,25,26]. In contrast, the specificity of hemin binding to G-quadruplexes is significantly high. This specific binding is attributable to an electrostatic repulsion between the carboxyl groups of hemin and the phosphate groups of DNA, leading to inhibition of hemin binding to ssDNA and dsDNA. In order to overcome the electrostatic repulsion, the large π-planar of hemin can form π-π stacking interactions with G-quartets of G-quadruplexes despite the electrostatic repulsion [27].

**Figure 3:**
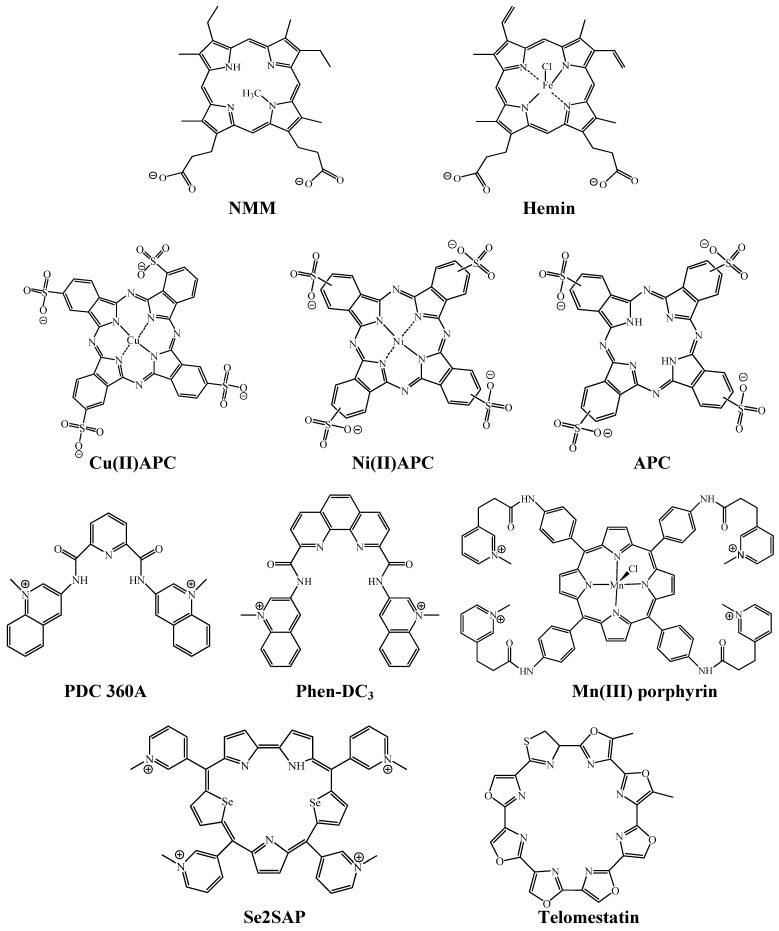
Chemical structures of anionic porphyrins (NMM and hemin), anionic phthalocyanines (Cu(II)APC, Ni(II)APC and APC), and other specific ligands for G-quadruplex (PDC 360A, Phen-DC_3_, Mn(III) porphyrin, Se2SAP and telomestatin).

G-quadruplexes play a role not only in the *in vitro* biosensing applications described above, but also in biological events in living cells [19,20,21,22,23,24,25,26]. G-quadruplexes, in particular those formed by human telomere sequences, have been investigated extensively. Human telomere DNA comprises tandem repeats of the sequence 5'-TTAGGG-3' with a 3' overhang of the G-rich strand at the ends of the chromosomes (Figure 2B) [28]. Telomere length shortening with each cell division limits the proliferative potential of normal somatic cells [28,29,30]. On the contrary, in 80%–85% of human tumor cells, functional telomerase elongates telomere DNA, which leads to immortal cell growth [31,32]. Human telomere G-quadruplex inhibits telomerase activity because the G-quadruplex can not function as a substrate for telomerase [33]; therefore, development of G-quadruplex-ligands that inhibit telomerase activity via induction or stabilization of the G-quadruplex has become a research area of great interest (Figure 2B) [19,20,21,22,23,24,25,26]. Ligands binding to the G-quadruplex have to be specific because any nonspecific binding to dsDNA in the genomic DNA could cause side effects. To date, it has been reported that various G-quadruplex-ligands such as bisquinolinium derivatives with a pyridodicarboxaminde core (PDC series) and phenanthroline dicarboxamide core (Phen-DC series), cationic pophyrin derivatives (Mn(III) porphyrin and Se2SAP), and telomestatin exhibit a G-quadruplex-over duplex-selectivity (Figure 3) [20]. Recent studies demonstrated that several phthalocyanines with functional groups including guanidinium group, quaternary ammonium group, and sulfo group are specific ligands of the G-quadruplex [24,25,26]. Notably, anionic phthalocyanines with sulfo groups bind to the G-quadruplex high specifically (Figure 3), which results in an efficient telomerase inhibition even in the presence of excess decoy dsDNA (Figure 2B) [25,26]. Thus, anionic phthalocyanines represent promising anticancer drug without side effects. Similar to hemin, the specificity of anionic phthalocyanines is attributable to an electrostatic repulsion with dsDNA. 

In this review, we will focus our attention on the specific binding of anionic porphyrins and phthalocyanines to G-quadruplexes, and discuss recent studies with a view to elucidating the binding mechanism and various applications utilizing the complex’s specific binding property. We will also briefly describe the implications of some other interesting applications.

## 2. Hemin-G-quadruplex Interaction for *in Vitro* Applications

Hemin, which is an anionic porphyrin with two carboxyl groups (Figure 3), is used as a cofactor for a variety of enzymes such as catalases, peroxidases and monooxygenases in living cells. In the late 1990s, it was discovered that some G-quadruplexes bind to hemin and the complexes show peroxidase-like activity [27]. Importantly, no DNA structures than other the G-quadruplex has such properties. Moreover, the peroxidase-like activity depends on G-quadruplex polymorphisms [27,34]. Thus, the studies on the relationships among DNA structure, binding property of hemin and the peroxidase-like activity have been conducted extensively. Furthermore, many applications utilizing the hemin-G-quadruplex peroxidase activity have been reported recently. Here, we discuss recent advances based on these studies.

### 2.1. Mechanism of Induction of Peroxidase-Like Activity

**Figure 4:**
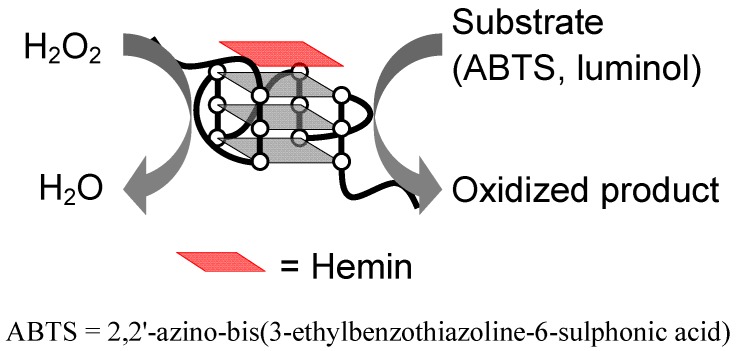
Peroxidase-like reaction of hemin-G-quadruplex.

In 1996 Sen’s group conducted an interesting investigation to find DNA aptamers for *N*-methylmesoporphyrin IX (NMM) (Figure 3) using an *in vitro* selection (Systematic Evolution of Ligands by Exponential enrichment, SELEX) method [35]. Surprisingly, the authors found that some G-rich DNAs including PS2 and PS5 bind to NMM despite the anionic properties of NMM (Table 1) [35]. More importantly, they showed that the G-rich DNAs are more than just NMM aptamers. NMM is a stable transition-state analogue for porphyrin-metallation reactions, implying that the NMM aptamers catalyze porphyrin metallation. In fact, it was demonstrated that PS2- and PS5-related DNAs have the ability to catalyze metallation [36,37]. Furthermore, since Sen’s group found that hemin, which is an anionic porphyrin utilized as a cofactor of peroxidase in nature, also binds to the NMM aptamers, the authors investigated the peroxidase-like activity of hemin-PS2.M and -PS5.M (Table 1) [27]. The investigation demonstrated that the observed catalytic velocity (*V*_obs_) of PS2.M-hemin was 250 times greater than that of hemin alone when 2,2'-azino-bis(3-ethylbenzothiazoline-6-sulphonic acid) (ABTS) was used as a substrate in the presence of H_2_O_2_ (Figure 4) [27]. Interestingly, K^+^ was fundamental for the activity of PS2.M-hemin although Na^+^ and Mg^2+^ inhibited the activity [27]. The result implies that the catalytically-active form of PS2.M is a specific G-quadruplex structure that is stabilized by K^+^. The characteristic ability of hemin to bind specifically to the G-quadruplex is a key point for many applications described below.

**Table 1:** G-rich sequences of G-quadruplexes with peroxidase-like activity.

Name	Sequences (5'→3')
PS2	TTGCC TAACC GTGAA GGTAA AACGA TTTAG TCAAA CGTGG GAGGG CGGTG GTGTT GACTG ATCGA TTTTA TTCCA
PS5	GTGTC GAAGA TCGTG GGTCA TTGTG GGTGG GTGTG GCTGG TCCGA TCCGC GATCT GCTGA CGCTG GTTAG GT
PS2.M	GTGGG TAGGG CGGGT TGG
PS5.M	GTGGG TCATT GTGGG TGGGT GTGG
CatG4	TGGGT AGGGC GGGTT GGGAA A
TBA	GGTTG GTGTG GTTGG
AGRO100	GGTGG TGGTG GTTGT GGTGG TGGTG G

**Figure 5:**
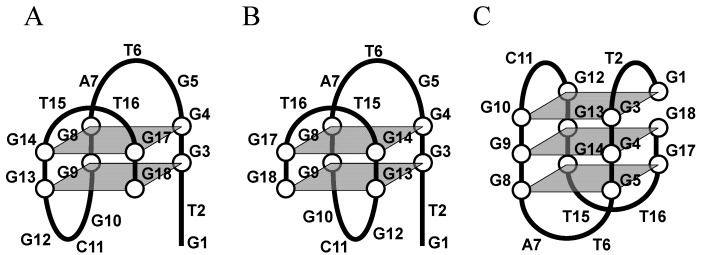
Proposed structures of the PS2.M G-quadruplex in 20 mM KCl (**A** and **B**) [38] and in 140 mM NaCl (**C**) [39].

**Figure 6:**
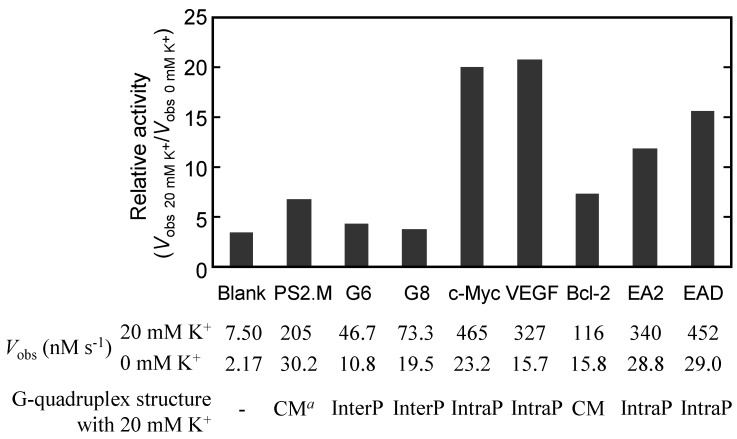
Enhancement of peroxidase-like activity by K^+^ [41]. Relative activity values were obtained from the equation (*V*_obs_ of each G-quadruplex with hemin in a buffer containing 20 mM K^+^)/(*V*_obs_ of each G-quadruplex with hemin in a buffer containing 0 mM K^+^), and *V*_obs_ values are from ref. 41. G-quadruplex structures were determined by CD spectra [41]. Blank means hemin with no G-quadruplex. The sequences of G-quadruplexes are as follows: G6 is 5'-T_4_G_6_T_4_-3', G8 is 5'-T_4_G_8_T_4_-3', c-Myc is 5'-TGAG_3_TG_4_AG_3_TG_4_A_2_-3', VEGF is 5'-(G_3_C)_2_CG_5_CG_3_-3', Bcl-2 is 5'-G_3_CGCG_3_AG_2_A_2_G_5_CG_3_-3', EA2 is 5'-CGAGG(TG_3_)_3_A-3', and EAD is 5'-C(TG_3_)_4_A-3'. CM, InterP, and IntraP mean “coexisting of parallel and antiparallel G-quadruplexes or mixed type hybrid G-quadruplex”, “intermolecular parallel G-quadruplex”, and “intramolecular parallel G-quadruplex”, respectively. ^a^ The CD spectrum is very similar to the typical one for a parallel G-quadruplex.

The pioneering finding of the peroxidase-like activity of hemin-G-quadruplex has stimulated studies into the mechanisms of action, in particular the relationship among structural polymorphisms of G-rich sequences, the binding property of hemin and the peroxidase-like activity. However, the detail of the PS2.M structure remains to be completely elucidated and several models of the structure have been proposed to date [38,39,40,41]. Since the results of methylation protection assays indicate that G9, G17, and G18 in PS2.M are essential for forming G-quartets, and G10 and G12 participate in hemin binding, Sen’s group proposed two models of active forms of PS2.M: unimolecular antiparallel G-quadruplexes with two G-quartets including G9, G17, and G18; and two top and bottom protrusive loops including G10 and G12 (Figure 5A and 5B) [38]. However, it is impossible to determine the orientation of each strand in the G-quadruplex by methylation protection assay although the assay is an effect tool to demonstrate essential bases involved in G-quadruplex formation and hemin binding. Circular dichroism (CD) spectra give structural information on G-quadruplexes including the strand orientation. By employing CD analysis, it was found that, in the presence of K^+^, PS2.M forms an antiparallel G-quadruplex initially at optical concentrations, but with additional time or at higher initial strand concentrations an increasing amount of a multistranded parallel G-quadruplexes appear [39]. In contrast to K^+^, 140 mM Na^+^ or 140 mM Pb^2+^ caused a stable unimolecular antiparallel G-quadruplex of PS2.M (Figure 5C) [39]. Shangguan’s group also studied the structure and the peroxidase-like activity of PS2.M using CD analysis [41]. The authors suggested that, in the presence of 150 mM Na^+^ and 20 mM K^+^, PS2.M forms both parallel and antiparallel G-quadruplexes, or a hybrid type of G-quadruplex [41]. However, the CD spectrum of PS2.M observed by the authors is very similar to the spectrum for a parallel G-quadruplex. Thus, most of the G-quadruplexes under these conditions may be parallel G-quadruplexes. Furthermore, the authors also showed that the presence of as little as 20 mM K^+^ enhanced peroxidase-like activity of PS2.M with hemin (Figure 6). According to the results with PS2.M, K^+^ may be important for the formation of a parallel G-quadruplex of PS2.M, leading to higher peroxidase-like activity with hemin.

The relationship between the G-quadruplex structure and the peroxidase-like activity has also been investigated using other G-quadruplex-forming sequences [40,41,42]. As shown by the activity dependency on the coexisting cation, G-rich sequences forming a parallel G-quadruplex in the presence of K^+^ showed higher peroxidase-like activity (Figure 6) [40,41]. Not only the structural orientation, but also the structure of loop region are important for hemin binding and the enzymatic activity [41]. This is attributable as follows: for the unimolecular G-quadruplex, distribution of the loops on the sides of the G-quadruplex allows hemin end-stacking to G-quartets, but for the antiparallel G-quadruplex protrusion of the loops over the top and bottom G-quartets inhibits end-stacking. For the multistranded parallel G-quadruplexes formed by T_4_G*_n_*T_4_ (*n* = 6 or 8), T4 termini of the strands hinders the end-stacking of hemin [41]. Systematic studies on the structure-function relationship of d(G_2_T*_n_*)_3_G_2_ and d(G_3_T*_n_*)_3_G_2_ (*n* = 1 − 4) also demonstrated that the parallel G-quadruplexes in the presence of 100 mM K^+^ have high peroxidase-like activity although the antiparallel G-quadruplexes in the presence of 100 mM Na^+^ have little activity [42].

These studies with various G-quadruplexes strongly suggest that the peroxidase-catalytically active form is the parallel conformation induced by K^+^. These findings are important for the biosensing application utilizing hemin-G-quadruplex peroxidase because conformational switching between the catalytically inactive and the active forms of the G-quadruplex is crucially important for this application. In the next section we will discuss representative applications, mainly DNA detection, utilizing the conformation switch.

### 2.2. DNA Detection

Hemin-G-quadruplex peroxidases have been widely applied to biosensing for various target molecules and ions. In particular, hemin-G-quadruplex peroxidases have been widely applied for sequence-specific DNA detection.

#### 2.2.1. DNA Detection Utilizing Conformational Change of Probe DNA

**Figure 7:**
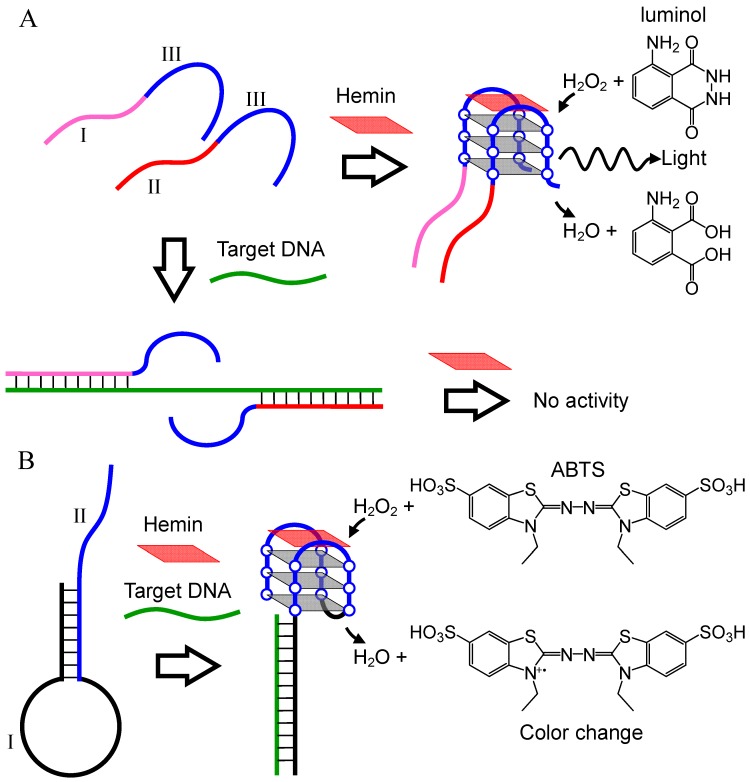
DNA detection techniques based on the G-quadruplex formed by two probe DNAs (**A**), and the conformational change of a stem-loop type probe DNA into G-quadruplex (**B**).

In 2004, Willner’s group first reported a DNA detection technique using the hemin-G-quadruplex peroxidase. The technique utilizes two probe DNAs, which contain a complementary sequence (I, II) to the target DNA and a G-quadruplex forming sequence (III) (Figure 7A) [43]. In the absence of the target DNA, the catalytically-active G-quadruplex is formed by the two probe DNAs via self-assembly, although the binding of the probe DNAs to the target DNA inhibits G-quadruplex formation. The catalytically active G-quadruplex with hemin oxidizes luminol in the presence of H_2_O_2_, to chemiluminescence. Based on this scheme, the detection of the target oligonucleotide was possible to a detection limit of 0.6 μM (Table 2). The same group further developed the stem-loop type probe DNA with overhanging ssDNA at the end (Figure 7B) [44]. The stem-loop DNA sequence contains a complementary sequence (I) to the target DNA in the loop and a G-quadruplex forming sequence (II) in the stem and the overhanging part (Figure 7B). The hybridization between the target DNA and the loop DNA unfolds the stem-loop structure, leading to formation of a catalytically-active G-quadruplex involving the stem and DNA overhang. This sensing characteristic allows the detection of a target oligonucleotide with a detection limit of 0.2 μM using the luminol-H_2_O_2_ system (Table 2).

#### 2.2.2. Signal Amplification by Polymerase Reaction

**Figure 8:**
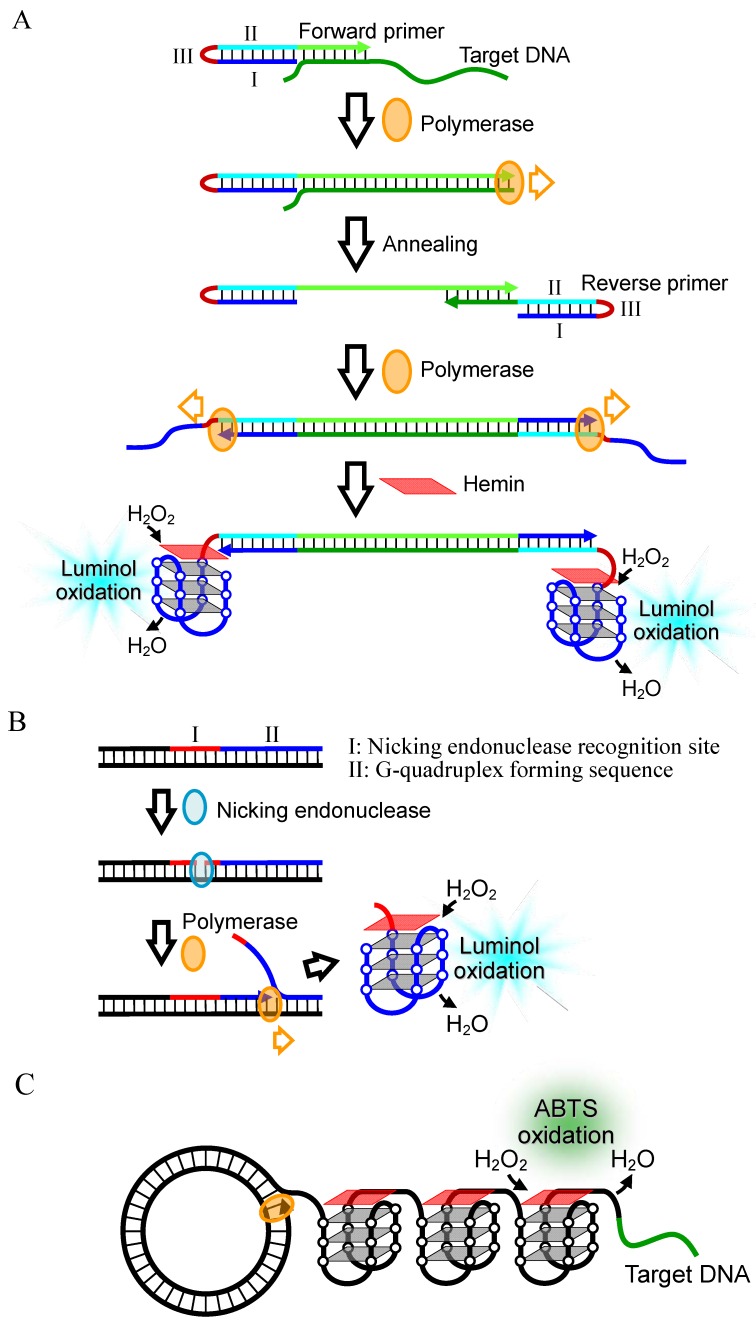
(**A**) DNA detection technique based on G-quadruplex amplification via PCR; (**B**) G-quadruplex amplification technique with nicking and strand displacement polymerase extension reactions, and (**C**) DNA detection technique based on G-quadruplex amplification via RCA.

Since sensitive detection of DNA requires many copies of catalytically-active G-quadruplex, some DNA detection techniques utilizing amplification of the G-quadruplex by polymerase reaction have been proposed. Here the long target DNA is amplified by polymerase chain reaction (PCR) using primers with a 5' tail sequence, which includes a G-quadruplex forming sequence (I) tethered to the cytosine-rich (C-rich) complementary sequence (II) via an oxyethyleneglycol bridge (III) (Figure 8A) [45]. Before the PCR amplification occurs, the G-quadruplex forming sequence hybridizes with the C-rich complementary sequence. In the presence of the target DNA, however, the amplification reaction produces the replicated dsDNA structure including the target DNA sequence and the C-rich complementary sequence, which results in the release of the G-quadruplex forming sequence. This leads to the formation of a catalytically-active G-quadruplex with hemin. The G-quadruplex increases with the PCR amplification. The M13 phage DNA at a concentration of 1.2 aM was detectable with the luminol-H_2_O_2_ system after 30 PCR cycles (Table 2). The detection limit is the highest among those of DNA detection techniques utilizing hemin-G-quadruplex peroxidase. The authors further developed an isothermal technique for G-quadruplex amplification, in which nicking of the DNA by nicking endonuclease, and strand displacement polymerase extension occur repetitively (Figure 8B) [46]. Utilizing this technique with the ABTS-H_2_O_2_ system or luminol-H_2_O_2_ system, M13 phage DNA was detected up to a detection limit of 10 fM (Table 2) [46]. In addition, another isothermal DNA detection technique utilizing the rolling circle amplification (RCA) method, in which the target DNA functions as the primer and the catalytically-active G-quadruplex is amplified, was developed (Figure 8C) [47]. As little as 1 pM of the target DNA was detectable by analyzing peroxidase-like activity of the G-quadruplex with the ABTS-H_2_O_2_ system (Table 2). 

**Table 2:** DNA detection techniques using peroxidase-like activity of G-quadruplex with hemin.

Ref.	Principle	Target DNA	Indicator	Detection Limit	Experimental Conditions
[43]	Figure 7A	5'-TTGAG CATGC GCATT ATCTG AGCCA GTACC GAATC G-3'	Luminol	0.6 μM	25 mM HEPES buffer, 20 mM KCl, 200 mM NaCl, 0.5 mM luminol, 30 mM H_2_O_2_, pH 9.0 *^a^*
[44]	Figure 7B	5'-AATGG CAGCA ATTTC ACCAG TACTA CAGTT AAGGC-3'	ABTS	0.2 μM	0.1 M Tris buffer, 20 mM MgCl_2_, 0.43 μM hemin, 3.2 mM ABTS, 3.2 mM H_2_O_2_, pH 8.1
[45]	Figure 8A	M13 phage DNA	Luminol	1.2 aM	25 mM HEPES buffer, 20 mM KCl, 200 mM NaCl, 1 nM hemin, 0.5 mM luminol, 30 mM H_2_O_2_, pH 9.0
[46]	Figure 8B	M13 phage DNA	ABTS or luminol	10 fM	Colorimetric measurements (ABTS) : 25 mM HEPES buffer, 20 mM KCl, 200 mM NaCl, 0.4 μM hemin, 182 μM ABTS, 44 μM H_2_O_2_, pH 7.4; Chemiluminescence measurements (luminol): 25 mM HEPES buffer, 20 mM KCl, 200 mM NaCl, 1 nM hemin, 0.5 mM luminol, 30 mM H_2_O_2_, pH 9.0
[47]	Figure 8C	5'-CTCAC ACGAA TTCAT CTGAC-3'	ABTS	1 pM	15 mM Tris-HCl, 3 mM MgCl_2_, 1 mM (NH_4_)_2_SO_4_, 0.01 mg/mL BSA, 0.005% Triton X-100, 0.5 μM hemin, 1.17 mM ABTS, 2.82 mM H_2_O_2_
[48]	Figure 9A	5'-CCTCC CGGTG TTCGA TCC-3'	Luminol	76 aM	*^b^*
[49]	Figure 9B	5'-TCGAA TAAGC ACTGA GGT-3'5'-ATAAA TTGCC AAGAT GAT-3'5'-TATCA ATACT CCCCC AGG-3'	CRET (luminol)	10 nM	25 mM HEPES buffer, 20 mM KNO_3_, 200 mM NaNO_3_, 10 nM hemin, pH 9.0 *^c^*
[50]	Figure 9C	5'-TTGAG CATGC GCATT ATCTG AGCCA GTACC GAATC G-3'	Luminol	100 pM	25 mM HEPES buffer, 20 mM KCl, 200 mM NaCl, 0.5 mM luminol, 30 mM H_2_O_2_, pH 9.0
[51]	Figure 10	5'-TCGAA TAAGC ACTGA GGT-3'	Electrocatalytic cathodic current	1 pM	10 mM HEPES buffer, 50 mM KCl, 150 mM NaCl, 1 μM hemin, 1 mM H_2_O_2_, pH 7.2

^a^ The chemiluminescence measurement was performed by adding 3.3 mL of the measurement buffer to 15 μL of the sample solution containing the probe DNAs, the target DNA, and 12 μM hemin; ^b^ 200 μL of 10 mM phosphate buffer (pH 7.4) containing 10 mM NaCl and Fe_3_O_4_-Au nanoparticle modified with the probe DNA was reacted with 100 μL of the target DNA. Upon incubation, 1 μL of nicking endonuclease was added to the solution. After incubation, the resulting mixtures were heated to deactivate the nicking endonuclease, followed by the addition of hemin (10 nM), luminol (1 μM). The chemiluminescence reaction was triggered by adding 100 μL of H_2_O_2_ (0.75 mM); ^c^ The CRET measurement was performed by adding 50 μM luminol and 300 μM H_2_O_2_ to the measurement buffer containing samples.

#### 2.2.3. Combination with Nanoparticle

**Figure 9:**
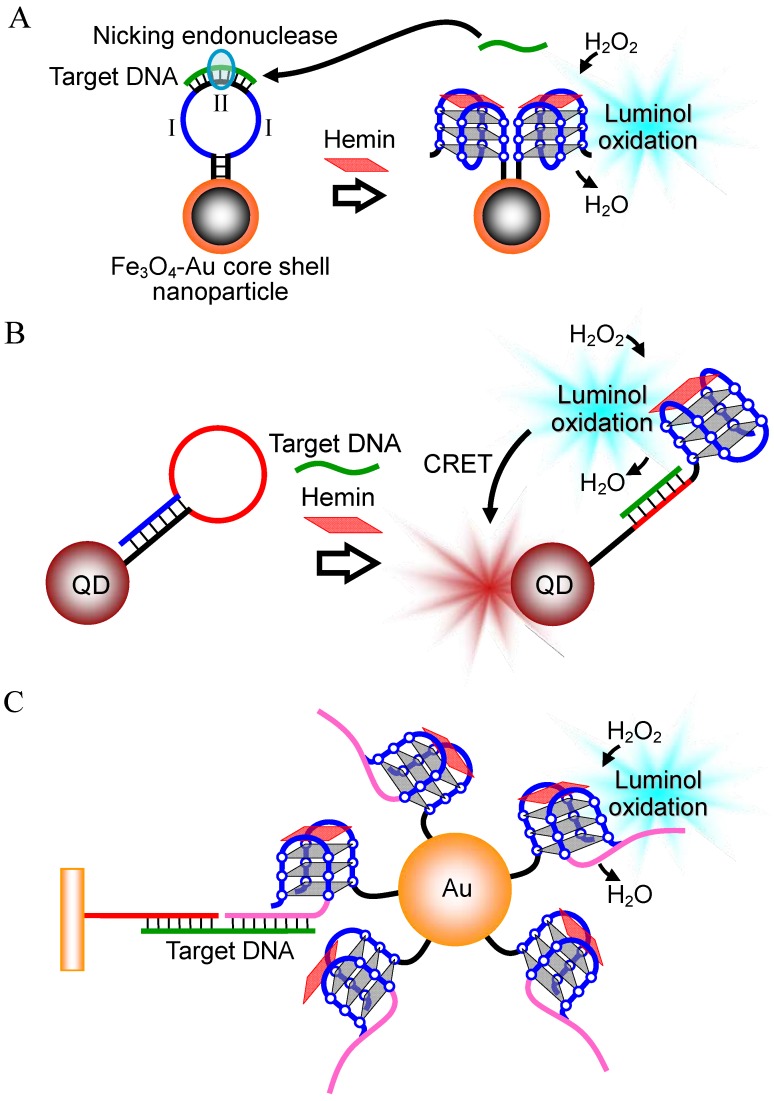
DNA detection techniques utilizing the Fe_3_O_4_-Au core shell nanoparticle (**A**); QDs (**B**), and Au nanoparticles (**C**).

Novel DNA detection techniques utilizing a probe DNA tethered to nanoparticles have been developed. Zhang’s group immobilized both ends of a stem-loop type probe DNA onto a Fe_3_O_4_-Au core shell nanoparticle (Figure 9A) [48]. The loop of the probe DNA contains two G-quadruplex forming sequences (I), and between the sequences a complementary sequence (II) to the target DNA is designed. Moreover, the loop hybridizing with the target DNA is nicked by a nicking endonuclease. The nicking of the loop DNA generates two catalytically-active G-quadruplexes and releases the target DNA. The released target DNA binds to the loop of another probe DNA, which initiates the same reaction described above. The repetitive successive reactions generate a number of copies of the G-quadruplexes. According to the principle, the target DNA at a concentration of 76 aM is detectable with the luminol-H_2_O_2_ system (Table 2).

Chemiluminescence resonance energy transfer (CRET) of the chemiluminescence from oxidized luminol to quantum dots (QDs) was further combined for DNA detection (Figure 9B) [49]. The probe DNA used to demonstrate this principle is a stem-loop type DNA, which is immobilized to CdSe/ZnS QDs. The hybridization of the target DNA with the loop region unfolds the stem-loop structure, which results in formation of the catalytically-active G-quadruplex. Luminol chemiluminescence produced by the G-quadruplex peroxidase-like activity excites the CdSe/ZnS ODs, leading to a CRET signal. Designing of probe DNA sequences and regulating the emission wavelength of CdSe/ZnS QDs depending on the target DNA sequence allows the detection of different target DNAs simultaneously with different CRET signals (Table 2). Willner’s group also modified the Au nanoparticle with a probe DNA, which contains a complementary sequence to the target DNA and a catalytically-active G-quadruplex (Figure 9C) [50]. The functionalized Au nanoparticles are gathered to a gold substrate via the target DNA. The detection limit for analyzing the activity of the G-quadruplex on the gold substrate with the luminol-H_2_O_2_ system is 100 pM (Table 2).

#### 2.2.4. Electrochemical DNA Detection

**Figure 10:**
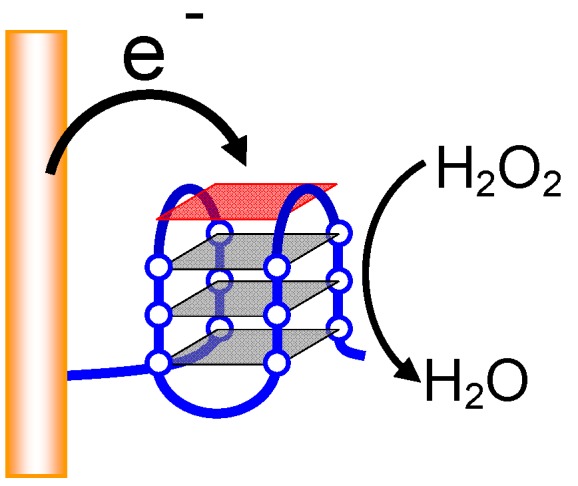
Electrochemical DNA detection in solid phase based on direct electron transfer from an Au electrode to the G-quadruplex.

For almost all DNA detection techniques using hemin-G-quadruplex peroxidase, ABTS and luminol are used as an indicator, leading to optical detection of the target DNA. However, in general an optical detection technique requires expensive and large equipment such as UV-Vis and fluorescent spectrophotometers. Li’s group, then, first demonstrated that direct electron transfer can occur between a hemin-G-quadruplex and an electrode in the presence of H_2_O_2_ using the pyrolytic graphite electrode coated with hemin-G-quadruplex-cetyltrimethylammonium bromide (CTAB) film [52]. After the findings, Willner’s group detected target DNA utilizing the direct electron transfer from an electrode to the hemin-G-quadruplex (Figure 10) [51]. They immobilized a stem-loop type probe DNA on an Au electrode. The loop sequence is complementary to the target DNA and the stem part is dsDNA, which is formed by a G-quadruplex forming sequence and the C-rich complementary sequence. Then, hybridization of the target DNA to the loop opens the stem-loop structure, leading to formation of the catalytically-active G-quadruplex. Finally, the electrocatalytic cathodic currents generated by theG-quadruplex in the presence of hemin and H_2_O_2_ can be observed. The currents depend on the target DNA concentration, and the detection limit of the target DNA is 1 pM (Table 2). Electrochemical detection techniques usually require inexpensive and smaller detection devices compared with optical systems. Furthermore, direct electron transfer does not need a substrate for hemin-G-quadruplex peroxidase. Thus, the technique should allow for an inexpensive and portable DNA sensor to be used anywhere.

### 2.3. Biosensors for Various Targets

#### 2.3.1. Metal Ions

**Figure 11:**
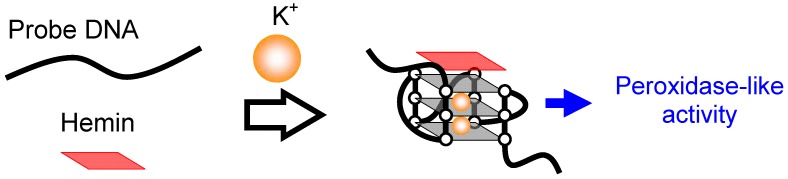
Common principle for detection of K^+^ using the hemin-G-quadruplex.

Metal ions play roles in biological systems and the concentrations must be maintained within a certain range. For example, a severe shortage of K^+^ in body fluids may cause a potentially fatal condition known as hypokalemia; in contrast, excess of K^+^ may increase the risk of high blood pressure and stroke [53,54]. Heavy metal ions including Ag^+^, Cd^2+^, Hg^2+^ and Pb^2+^ usually act as highly toxic environmental pollutants and have severe adverse effects on human health [55,56]. Thus, a measurement technique for metal ion concentration is essential. Conventional methods including ion chromatography, ion-selective electrodes and flame atomic adsorption spectrometry are complex and time-consuming; techniques that are simple, with short detection times and operation convenience are in demand. Metal ions critically affect conformation and thermal stability of G-quadruplexes, which enables the detection of the metal ions. K^+^ is one of the most studied coordination metal ions in G-quadruplexes. K^+^ enhances the peroxidase-like activity of various G-quadruplexes by changing and stabilizing the structures [27,40,41,42]. The analysis of change in activity as a function of K^+^ enables the measurement of K^+^ concentrations (Figure 11) [54,57,58]. Pb^2+^ and Tb^3+^ also are known to promote compact G-quadruplexes and influence the peroxidase-like activity in the presence of hemin. Based on this principle, several Pb^2+^ and Tb^3+^ detection techniques have been developed [59,60,61].

**Figure 12:**
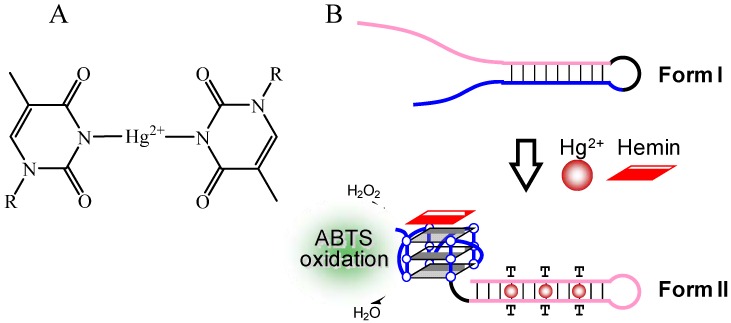
(**A**) Chemical structure of the T-Hg^2+^-T base pair; (**B**) Hg^2+^ detection is based on a conformational change from a stem-loop type probe DNA with long ssDNAs to a G-quadruplex with dsDNA containing T-Hg^2+^-T base pairs.

**Figure 13:**
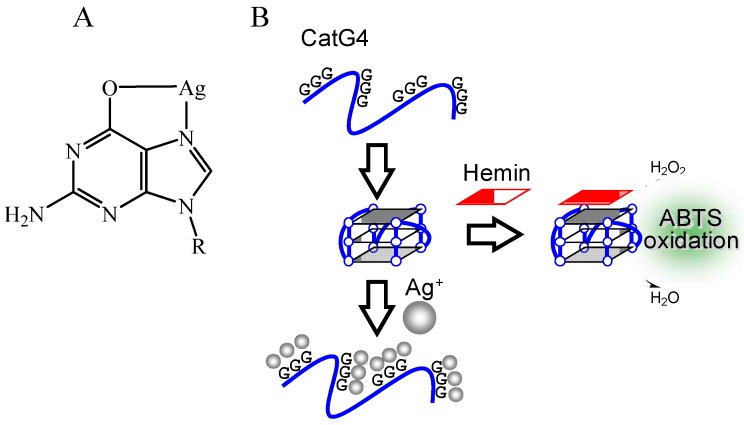
(**A**) Chemical structure of G-Ag^+^ interaction; (**B**) Ag^+^ detection based on disruption of the G-quadruplex by G-Ag^+^ interaction.

Unlike the principle for the detection of K^+^ and Pb^2+^, the binding of Hg^2+^ to thymine (T) base is usually utilized in the detection of Hg^2+^ (Figure 12A) [62,63,64,65]. For example, Shen’s group developed a probe DNA that forms a stem-loop structure with two long ssDNAs at both ends (Form I) in the absence of Hg^2+^ and hemin (Figure 12B) [65]. The stem-loop structure is changed to the G-quadruplex with dsDNA containing three T-Hg^2+^-T base pairs (Form II) by the addition of Hg^2+^ and hemin. Peroxidase-like activity of the G-quadruplex with hemin enables the selective detection of Hg^2+^ over other metal ions. The binding of Ag^+^ to DNA bases can also be exploited for the detection of Ag^+^ [66,67]. For example, the binding of Ag^+^ to G base disrupts the G-quadruplex structure of CatG4 (Table 1), which has peroxidase-like activity with hemin (Figure 13) [66]. Thus, the monitoring of the reduction of peroxidase depending on this disruption allows the detection of Ag^+^. A similar principle for the detection of Hg^2+^ as shown in Figure 12 was also applied to the detection of Ag^+^ [67]. In the case of Ag^+^ detection, Form II contains dsDNA with two C-Ag^+^-C base pairs. In addition to the metal ion binding to natural bases, several artificial bases, which can bind strongly to some metal ions including Fe^3+^, Ni^2+^, Ag^+^ and Hg^2+^, have been developed [9,68,69,70,71,72,73]. The incorporation of these artificial bases into the probe DNA should not only improve the affinity and specificity but also expand the applications.

#### 2.3.2. Proteins

**Figure 14:**
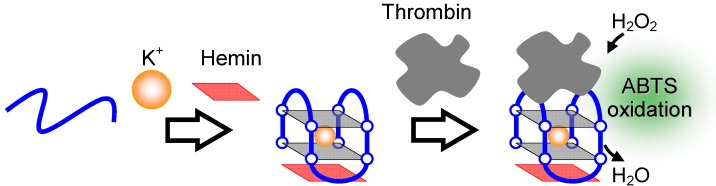
Detection of thrombin based on formation of a thrombin-TBA-hemin super complex.

Detection of proteins is invaluable for disease diagnoses including cancer diagnosis. In general, an immunoassay technique is utilized for detection. However, it is cumbersome, time-consuming and expensive to develop the correct antibody and difficult to keep the antibody stable for a long time. In addition, immunoassay procedures have some complex processes such as bound/free (B/F) separation. In order to overcome these drawbacks, some protein detection techniques using hemin-G-quadruplex peroxidase have been developed to date. A DNA aptamer for thrombin, which plays a role in blood coagulation by catalyzing the conversion of fibrinogen to fibrin in blood coagulation, forms a G-quadruplex structure [16,74,75]. The thrombin aptamer (TBA, Table 1) forms a complex with thrombin and hemin, and the complex shows higher peroxidase-like activity than the hemin-TBA complex alone (Figure 14) [76]. According to these findings, optical and electrochemical detection techniques for thrombin were developed [76,77].

**Figure 15:**
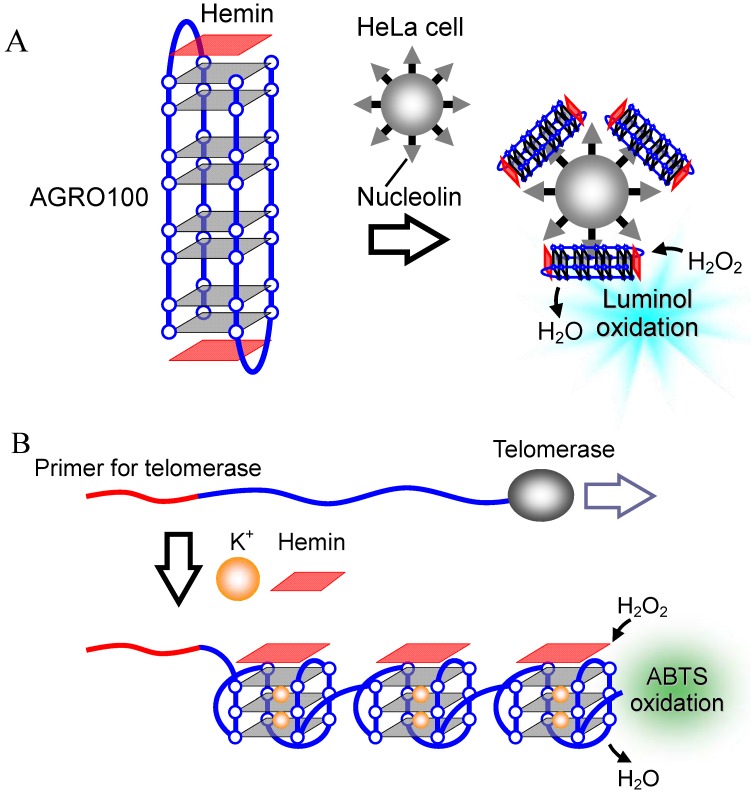
(**A**) Detection of HeLa cells based on hemin-G-quadruplex binding to nucleolin on the cell membrane; (**B**) Detection of telomerase activity based on a conformational change of telomerase reaction product into G-quadruplexes in long strands.

A DNA aptamer, AGRO100 (Table 1), for nucleolin, which is a protein marker expressed at high levels at the cell membrane surface of human cancer cells, also forms a G-quadruplex structure and exhibits significantly high peroxidase-like activity with hemin in the presence of K^+^ [78,79]. Based on the specific interaction between hemin-AGRO100 and nucleolin, HeLa cells are labeled with hemin-AGRO100, which allows detection of 6000 HeLa cells using the luminol-H_2_O_2_ system (Figure 15A) [80].

Telomerase, an enzyme involved in cancer development, can also be detected using hemin-G-quadruplex peroxidase-like activity [81,82,83,84]. Telomerase can elongate the telomere DNA, which is a repetitive G-rich sequence composed of 5'-(TTAGGG)_n_-3' [31,32]. The telomere DNA forms a G-quadruplex, resulting in the peroxidase-like activity of the telomere DNA with hemin (Figure 15B) [82,83].

## 3. Anionic Phthalocyanine-G-Quadruplex Interaction for *in Vivo* Applications

Like hemin, anionic phthalocyanines also have a large π-planar core with anionic functional groups. The properties of binding to human telomere G-quadruplex and consequent telomerase inhibition, which could prevent immortal growth of cancer cells, have been demonstrated quantitatively [25,26]. Here, we provide an overview of these properties and discuss the potential of anionic phthalocyanines as a novel class of anticancer drug.

### 3.1. G-Quadruplex-Ligands as Telomerase Inhibitors

Since the discovery that the human telomere G-quadruplex inhibits telomerase activity, development of G-quadruplex-ligands that inhibit telomerase activity via induction or stabilization of the G-quadruplex has become an area of great interest (Figure 2B) [19,20,21,22,23,24,25,26]. Many of the ligands developed to date contain a large π-planar structure and cationic functional groups in order to form strong π-π stacking and electrostatic interactions with G-quartets and phosphate groups on the G-quadruplex, respectively [19,20,21,22,23,24,25,26]. The most extensively studied G-quadruplex-ligand with such structural features is 5,10,15,20-tetra-(*N*-methyl-4-pyridyl)porphine (TMPyP4), which is a cationic porphyrin derivative composed of four modified pyrrol units and four cationic functional groups [19,20,21,22,23,24,25,26]. Based on results from UV titration experiments, NMR and photocleavage assays, Hurley’s group reported for the first time that TMPyP4 binds to the human telomere G-quadruplex [19]. They further demonstrated that TMPyP4 inhibits telomerase activity in a cell-free system [19]. In accordance with these results, TMPyP4 inhibits proliferation of various tumor cells including human pancreatic, breast and prostate carcinomas [85,86,87,88,89,90,91]. However, the positive charges carried by TMPyP4 also mediate non-specific interactions with dsDNA [20,22,23,25]. These non-specific interactions with dsDNA could cause cytotoxicity to normal cells and reduce ligand binding to human telomere G-quadruplexes, resulting in reduction of the telomerase inhibitory function [86]. Actually, the half-maximal inhibitory concentration (IC50) of TMPyP4 for human telomerase is 3.7 μM in the absence of dsDNA, but the addition of dsDNA completely eliminates this inhibitory effect [25]. Thus, a novel strategy for designing G-quadruplex-ligands with higher selectivity over dsDNA is absolutely imperative. One solution to this issue is introducing anionic functional groups into the larger π planar core that should be able to completely eliminate electrostatic interactions with the anionic phosphate groups of dsDNA, while the large π planar core would enhance the affinity of π-π stacking interactions with the G-quadruplex. Anionic porphyrins with carboxyl groups, NMM and hemin (Figure 3), are known to bind to the human telomere G-quadruplex with high selectivity over ssDNA and dsDNA [27,92,93]. Notably, it was recently demonstrated that some anionic phthalocyanines also can bind to the human telomere G-quadruplex specifically over dsDNA and ssDNA, and inhibit telomerase activity efficiently even in the presence of excess dsDNA [25,26]. 

### 3.2. Selective Binding of Anionic Phthalocyanines to the Human Telomere G-quadruplex

Incorporation of anionic functional groups into phthalocyanines is a promising strategy for eliminating non-specific binding to dsDNA. The capacity of an anionic copper phthalocyanine containing four sodium salt forms of sulfo groups (Cu(II)APC, Figure 3) to bind to the human telomere G-quadruplex and its capacity to inhibit telomerase activity have been investigated [25,26]. Based on visible absorbance titration experiments, Cu(II)APC bound to a human telomere G-quadruplex (5'-GGG(TTAGGG)_3_-3') with a dissociation constant (*K*_d_) of 42 μM in the presence of 100 mM KCl. On the contrary, little or no absorbance change was observed for ssDNA (5'-T_21_-3') or dsDNA (5'-AGAAGAGAAAGA-3'/5'-TCTTTCTCTTCT-3') (Table 3). These results indicate that Cu(II)APC can bind to the G-quadruplex with high selectivity over ssDNA and dsDNA. Notably, the *K*_d_ of Cu(II)APC for the G-quadruplex in the presence of excess decoy dsDNA, lambda DNA (a condition intended to mimic conditions in cell nuclei) was almost the same as that in the absence of lambda DNA (Table 3). Furthermore, to investigate the effect of a coordinating metal on anionic phthalocyanine binding to the G-quadruplex, binding assays were performed using nickel anionic phthalocyanine containing four sodium salt forms of sulfo groups (Ni(II)APC, Figure 3). Ni(II)APC also bound to the G-quadruplex and the *K*_d_ values in the absence and presence of lambda DNA were almost identical (Table 3). 

**Table 3:** Comparison of properties of anionic phthalocyanines and TMPyP4.

		*K*_d_/μM					IC50/μM	
		G-quadruplex ^a^ (−Lambda DNA)	ssDNA ^b^	dsDNA ^c^	G-quadruplex (+Lambda DNA)		−Lambda DNA	+Lambda DNA
Cu(II)APC		42	>250	>250	36		1.2	1.2
Ni(II)APC		56	>250	>250	71		1.9	1.4
APC		-	-	-	-		2.4	2.3
TMPyP4		0.7	-	-	No binding ^d^		3.7	No inhibition ^e^

^a^^–^^c^ The sequences of G-quadruplex, ssDNA, and dsDNA are 5'-GGG(TTAGGG)_3_-3', 5'-T_21_-3', and 5'-AGAAGAGAAAGA-3'/5'-TCTTTCTCTTCT-3', respectively. ^d^ No change in absorbance spectra of TMPyP4 depending on G-quadruplex was observed. ^e^ No inhibition of telomerase activity was observed in the presence of TMPyP4 up to 10 μM.

Similar to hemin, the selective binding property of Cu(II)APC and Ni(II)APC is attributable to the electrostatic repulsion between anionic groups of anionic phthalocyanines and anionic phosphate groups of dsDNA. In contrast, lambda DNA prevented TMPyP4 from binding to the G-quadruplex, which is thought to be due to nonspecific binding of TMPyP4 to dsDNA (Table 3).

### 3.3. Telomerase Inhibition by Anionic Phthalocyanines

**Figure 16:**
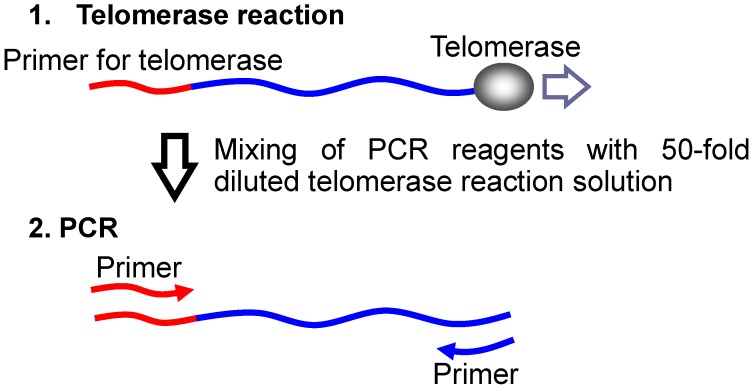
Schematic diagram of the two-step TRAP assay.

Its highly selective binding property suggests that Cu(II)APC can inhibit telomerase activity with or without excess dsDNA. Hence, the telomerase inhibitory effect was studied using a modified telomere repeat amplification protocol (TRAP), which comprises two reactions: a telomerase reaction in the presence of 63 mM KCl at 30 °C for 30 min, and a polymerase chain reaction (PCR) using a 50-fold diluted telomerase product as template (Figure 16) [25,26,94,95]. 

**Figure 17:**
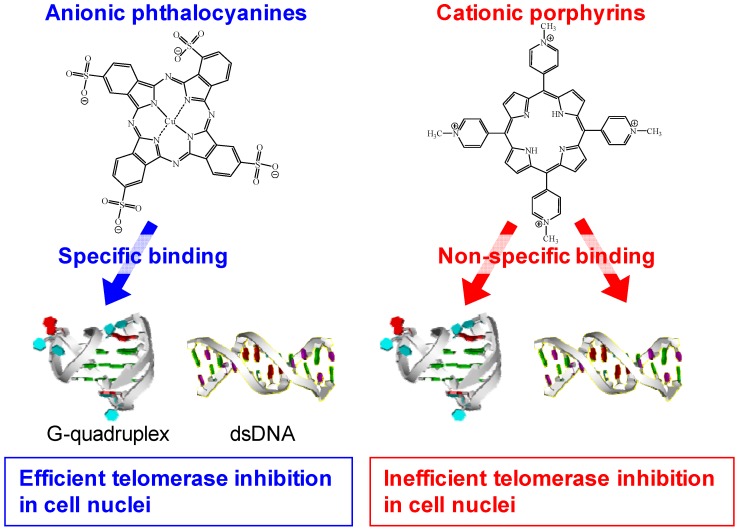
Schematic diagram of the properties of anionic phthalocyanines and cationic porphyrins for binding to the G-quadruplex and inhibiting telomerase activity.

The results from the TRAP assay were in accordance with the results of the binding studies; Cu(II)APC inhibited telomerase activity with an IC50 value of 1.2 μM in both the absence and presence of lambda DNA (Table 3). Furthermore, Ni(II)APC and metal-free anionic phthalocyanines containing four sulfo groups (APC, Figure 3) also inhibited telomerase activity in the absence and in the presence of lambda DNA. The IC50 values for Ni(II)APC in the absence and presence of lambda DNA were 1.9 μM and 1.4 μM, respectively, whereas those for APC in the absence and presence of lambda DNA were 2.4 μM and 2.3 μM, respectively (Table 3). These results indicate that the coordination metal has very little effect on anionic phthalocyanine-mediated telomerase inhibition. Based on these findings, it is possible to conclude that, even in the presence of excess genomic dsDNA in cell nuclei, anionic phthalocyanines could efficiently inhibit telomerase activity (Figure 17). On the contrary, TMPyP4 lost the telomerase inhibitory effect in the presence of lambda DNA, and the IC50 value was 3.7 μM in the absence of lambda DNA, indicating that TMPyP4 cannot exert an inhibitory effect in cell nuclei (Figure 17). To the best of our knowledge, no other anionic molecules have been studied to determine whether they inhibit telomerase. Large π-planar compounds with anionic groups, like anionic phthalocyanines, could represent a new starting-material for the development of G-quadruplex-ligands, probes for G-quadruplexes and telomerase inhibitors. 

## 4. Summary and Outlook

Most G-quadruplex ligands are π-planar compounds with cationic groups for forming π-π stacking interactions with G-quartets and electrostatic interactions with anionic phosphate groups of G-quadruplexes [19,20,21,22,23,24,25,26]. However, the cationic ligands also nonspecifically bind to ssDNA and dsDNA electrostatically [20,22,23,25]. This nonspecific binding is not suitable for *in vitro* and *in vivo* applications related to G-quadruplexes because ssDNA and/or dsDNA coexist and could prevent the interaction between ligand and G-quadruplex in most applications. On the other hand, anionic porphyrins, hemin and NMM, and anionic phthalocyanines bind to G-quadruplexes with high selectivity [25,26,27,92,93]. Specific G-quadruplex-hemin complexes further exhibit peroxidase-like activity. All biosensing techniques utilizing the properties of the hemin-G-quadruplex do not require antibodies, and some of them do not even require enzymatic activity. Thus, such techniques have many advantages including inexpensive production costs, short production times and easy storage of materials. In addition to biosensing, novel logic gates utilizing hemin-G-quadruplex peroxidase have been developed recently [96,97,98]. Such technologies may open a new avenue for future nanoelectronics research. Moreover, studies have shown that some molecules other than ABTS and luminol also function as substrates of hemin-G-quadruplex peroxidase [99,100,101,102,103]. This expansion of substrates may allow *in vivo* application of hemin-G-quadruplex peroxidase. For example, the reactive oxygen species (ROS) scavenging system using peroxidases is crucial for cell resistance because ROS plays a role in various severe diseases including cancer [104,105]. Thus, smart oligonucleotides, which can form catalytically-active G-quadruplexes and oxidize biomolecular substrates in response to cellular oxidative stress, are promising as a novel type of environmentally-responsive antioxidant.

For *in vivo* applications of the anionic ligand, the specificity of anionic phthalocyanines for the human telomere G-quadruplex over ssDNA and dsDNA is also essential for efficient telomerase inhibition in cell nuclei. Furthermore, recent bioinformatic studies have shown that many putative G-quadruplex-forming sequences in the genome are enriched in the promoter regions of oncogenes including *C-MYC*, *C-KIT*, *H-RAS* and *K-RAS* [20,22,23,24,106,107]. These bioinformatic studies strongly indicate that G-quadruplexes can influence carcinogenesis by modulating transcription of oncogenes. Importantly, some G-quadruplex ligands regulate the expression of these oncogenes by binding to G-quadruplexes in the promoter regions [20,22,23,24]. Thus, anionic phthalocyanines are also expected to bind to G-quadruplexes in the promoter regions of oncogenes and regulate their expression.

Finally, phthalocyanines also represent promising molecules in the area of organic electronics because of their physical properties. In particular, dye-sensitized solar cells using phthalocyanines have been attracting increasing attention [108,109]. However, it is difficult to control association state, orientation and localization of phthalocyanines in the functional films on the devices because of their aggregation property. Programmable DNA nanostructure technology may be a promising approach to solving this problem [9]. A recent study has shown that G-quadruplexes can be fabricated on DNA nanostructure scaffolds [110]. Therefore, the desired dispersion and localization of anionic phthalocyanines could be realized because of the specific binding to the G-quadruplexes on the scaffold. Thus, together, anionic phthalocyanines and G-quadruplexes will contribute not only to the biomedical field but also to the organic electronics field. 
